# An ontology-based rare disease common data model harmonising international registries, FHIR, and Phenopackets

**DOI:** 10.1038/s41597-025-04558-z

**Published:** 2025-02-08

**Authors:** Adam S. L. Graefe, Miriam R. Hübner, Filip Rehburg, Steffen Sander, Sophie A. I. Klopfenstein, Samer Alkarkoukly, Ana Grönke, Annic Weyersberg, Daniel Danis, Jana Zschüntzsch, Elisabeth F. Nyoungui, Susanna Wiegand, Peter Kühnen, Peter N. Robinson, Oya Beyan, Sylvia Thun

**Affiliations:** 1 Berlin Institute of Health at Charité – Universitätsmedizin Berlin, Berlin, Germany; 2 Institute for Biomedical Informatics, University Hospital Cologne, Cologne, Germany; 3 Medical Data Integration Center (MeDIC), University of Cologne, Faculty of Medicine and University Hospital Cologne, Cologne, Germany; 4 Department of Paediatrics, University Hospital Cologne, Cologne, Germany; 5 Department of Neurology, University Medical Center Goettingen, Goettingen, Germany; 6 Department of Medical Informatics, University Medical Center Goettingen, Goettingen, Germany; 7 Department of Pediatric Endocrinology and Diabetology, Charité University Hospital, Berlin, Germany; 8 Center for Chronically Sick Children, Charité Universitätsmedizin Berlin, Berlin, Germany; 9 Berlin Center for Rare Diseases - Charité University Hospital, Berlin, Germany; 10 Deutsches Zentrum für Kinder- und Jugendgesundheit (DZKJ), Partner Site Berlin, Berlin, Germany; 11 The Jackson Laboratory for Genomic Medicine, Farmington, Connecticut USA

**Keywords:** Genetics research, Computational models, Information technology, Genome assembly algorithms, Data processing

## Abstract

Although rare diseases (RDs) affect over 260 million individuals worldwide, low data quality and scarcity challenge effective care and research. This work aims to harmonise the Common Data Set by European Rare Disease Registry Infrastructure, Health Level 7 Fast Healthcare Interoperability Base Resources, and the Global Alliance for Genomics and Health Phenopacket Schema into a novel rare disease common data model (RD-CDM), laying the foundation for developing international RD-CDMs aligned with these data standards. We developed a modular-based GitHub repository and documentation to account for flexibility, extensions and further development. Recommendations on the model’s cardinalities are given, inviting further refinement and international collaboration. An ontology-based approach was selected to find a common denominator between the semantic and syntactic data standards. Our RD-CDM version 2.0.0 comprises 78 data elements, extending the ERDRI-CDS by 62 elements with previous versions implemented in four German university hospitals capturing real world data for development and evaluation. We identified three categories for evaluation: Medical Data Granularity, Clinical Reasoning and Medical Relevance, and Interoperability and Harmonisation.

## Introduction

Rare diseases (RDs), although individually rare, collectively affect a substantial portion of the global population, estimated to be over 260 million individuals worldwide. This corresponds to around 4% of the population and over 17 million individuals in Europe, where a disease is considered rare if it affects less than 5 in 10.000 individuals. RDs, also referred to as Orphan Diseases, are suspected to have a genetic cause in over 70% of cases^1^.

The identification of the diagnosis for rare disease (RD) patients is often a time-consuming process due to a lack of awareness in non-RD-specific medical care and the systematic underrepresentation of RDs in routine care. A further challenge in the diagnosis of RDs arises from classification problems with clinical manifestations that are attributed to common diseases, which often lead to misdiagnoses^2^. While it cannot be expected of physicians to recognise each of the over 10,000 RDs^3^, this limitation frequently leaves patients without a diagnosis^4^. Delayed diagnoses impede the application of suitable treatments and advancements of research into novel therapies^5^, which are presently scarce^6^. Further, delay can lead to adverse psychological effects on patients and their families^7^.

Interoperability is defined as the unambiguous interpretation and seamless exchange of (medical) data achieved by using internationally agreed-upon standards. In medical facilities, data are often captured in unstructured formats without the use of standard ontologies, formats, and coding systems. Thus, the resulting data are not interoperable, rendering them unsuitable for precise research. However, standardised medical records in reusable formats are required to reach accurate conclusions. This is valid for all medical fields but is more critical for RD research, where data are inherently scarce^8^.

Furthermore, diagnosing RDs requires the consideration of more specialised and profound clinical data, such as deep phenotyping^9^, genotyping and patient history^10^. Comprehensive, standardised medical records in interoperable formats are critical to effective care and research advancements in RDs. Unfortunately, RDs are not listed in most cases in the widely used International Statistical Classification of Diseases 10th Revision (ICD-10), leading to difficulties in identifying RDs in electronic medical records^11^.

The need for a common data model for RDs (RD-CDM) stems from the complexity of over 10,000 distinct RDs^3^, each requiring specific data for research and clinical care. An RD-CDM addresses the challenge of unifying and simplifying data collection and implementation across healthcare information systems. This enables standardised comparisons across clinical settings, enhancing the efficiency and efficacy of RD research. By establishing common guidelines, the model ensures that RD-specific data is accurately maintained and preserved for in-depth analysis, which is essential for understanding the unique aspects of each disease. Adhering to interoperability requirements enhances the quality, precision, reusability, and accessibility of data, bridging the knowledge gap between clinical routine and specialised RD data. Furthermore, multiple secondary uses can be supported, including local utilisation (e.g. on-site analyses), transfer to central repositories (e.g., registries), and federated approaches (e.g., distributed analyses)^12^.

An RD-CDM must emphasise several success factors to optimise its utility and impact. Its scope should balance the spectrum of data granularity, reflecting the variety of detail observed within the data model. A common denominator with sufficient clinical detail to support specialist-level research is essential. Simultaneously, the scope must remain limited to ensure straightforward implementation and usability. While expanding the CDM yields benefits, expansion should cease once these benefits no longer outweigh the costs. As this is a dynamic process, operational flexibility is vital to sustain effectiveness across diverse applications, use cases, and international contexts^12^. This flexibility also facilitates linkage to Domain-Specific Common Data Elements^13^, which is essential for more complex research or the European Reference Networks^12,14^.

In this work, we demonstrate the development and evaluation of a novel RD-CDM^15^ that complies with international requirements on interoperability. For this purpose, previous studies and RD-CDMs were examined, finding that previous efforts often did not fully meet interoperability requirements or were implemented on a limited scale. The methodology for the development and evaluation of our RD-CDM^15^ comprised several steps that were carried out simultaneously across multiple sites.

To provide a brief overview, the process included the development in our open-source GitHub^16^ repository, alongside its documentation (https://rd-cdm.readthedocs.io/en/latest/). The selection of elements was based on the Common Data Set by European Rare Disease Registry Infrastructure (ERDRI-CDS), with extensions inspired by previous data models^17–20^, Fast Healthcare Interoperability Resources (FHIR) base resources^21^ and the Phenopacket Schema of the Global Alliance for Genomics and Health^22^. The extensional concepts were clinically evaluated to balance the model’s scope and granularity. All data elements and value sets were encoded using established ontologies to enhance semantic interoperability across the selected models and data standards. Finally, we implemented our RD-CDM^15^ in four German university hospitals, capturing real world data of various RDs in the Research Electronic Data Capture web application (REDCap). All steps and the rationale behind them are described in detail in the Methods section.

We identified three categories to evaluate our RD-CDM, further elaborated in the comprehensive Evaluation subsection of the Results. In detail, the subsections examine the (1) Medical Data Granularity within our model from a clinical perspective, discuss factors influencing the (2) Medical Reasoning and Clinical Relevance, and explore the advantages, limitations, and challenges related to (3) Interoperability and Harmonisation. To ensure consistency throughout the article, the Discussion section also follows the categories outlined in the Evaluation subsection. As this work is not a part of the clinical study, we do not elaborate on capturing and interpreting the real patient data. However, we emphasise how the implementations contributed to the clinical perspective of our methodological approach in developing and evaluating the RD-CDM.

Our RD-CDM, version 2.0.0^15^, can potentially enhance RD data quality and precision for clinical care and research. However, RD-specific data elements require extensive encoding to meet interoperability requirements, which requires further collaboration and work. Considering this is a non-balloted extension of the ERDRI-CDS and recognising the complexity of this approach, we emphasise the need for international collaboration and coordination to establish consensus-based RD-CDMs.

Ultimately, we would like to demonstrate that achieving better interoperability of medical data on rare diseases will lead to a positive and quantifiable improvement in identifying and treating such conditions. At present, we are not at a stage where we can fully leverage the benefits of the newly established data model. However, this work aims to demonstrate that the newly established data model enhances interoperability by supporting a wide range of use case scenarios and harmonising data standards essential for all rare diseases. The specifications defined in our model can help other RD researchers implement it within any hospital information system to generate FHIR resources, Phenopackets for analysis, and registry data compliant with international requirements, such as those of the European Reference Networks. In future, this could support the creation of sustainable frameworks around existing hospital information systems, delivering interoperability directly to clinicians.

This work can be considered as a proof of concept for developing and utilising RD-CDMs based on interoperability standards, as well as a baseline model for defining country-specific adaptations. We encourage further collaboration and wide implementation in various countries and healthcare information systems to sustainably improve the precision and quality of RD data internationally. By adhering to interoperability requirements, this approach can help bridge the knowledge gap between routine care data and specialised data necessary for meaningful RD care and research. Therefore, our analysis and insights on medical data granularity, medical reasoning, and harmonisation can be highly valuable for the international RD community.

### Background

The following subsection provides an overview of all technical terms and foundational concepts used in the tables below. We introduce abbreviations, key technologies, and schemas regarding the development and evaluation of our RD-CDM, as depicted in Tables 1–3. Further, we expand on previous RD data sets and models, common data elements, and CDMs. We then introduce the RD common data sets and models our work is based on.

**Table 1:** Definitions of FAIR principles and the semantic and syntactic interoperability layers including ontologies, terminologies, and respective references (Ref.).

Name (Abbreviation)	Definition	Ref.
FAIR Data Principles	Designed to improve the automated discovery and usability of data by machines while facilitating its reuse by individuals (Findable, Accessible, Interoperable, Reusable).	^78^
Interoperability	The ability of different systems, applications, or devices to connect and communicate in a coordinated manner without requiring effort from the end user.	^79^
Syntactic Interoperability	The ability of systems to communicate through compatible formats and protocols, such as JSON files, a format for sharing data in key-value pairs and arrays using human-readable text.	^79^
Semantic Interoperability	Ensures a shared and precise interpretation of (medical) data. Uniform data structuring and coding within ontologies, terminologies, and classifications enhance machine-to-machine communication, improving accuracy and outcomes.	^79^
Terminologies	A specific vocabulary used in healthcare communication, information exchange and documentation aspects, encompassing a set of preferred or official terms, whether as a systematic nomenclature supported by a centralized body or as common usage within a specific community of practice.	^80^
Ontologies	A controlled terminology with semantic relationships between concepts and a description logic for computational readability, provides formal and explicit representations of concepts and relationships within an area of healthcare to facilitate the organisation, integration, and exchange of knowledge across healthcare systems and applications.	^80^

**Table 2:** List of ontologies and terminologies used in the developed rare disease common data model, detailing their definitions and references (Ref.).

Name (Abbreviation)	Definition	Ref.
Human Phenotype Ontology (HPO)	A standardised vocabulary of phenotypic abnormalities providing a global standard for describing disease traits.	^81^
Sequence Ontology (SO)	Cohesive and standardised vocabulary for genomic annotation components, enhancing the sharing, analysis, and handling of genomic information.	^82^
International Statistical Classification of Diseases and Related Health Problems, 10th & 11th Revision (ICD-10, ICD-11)	Used for documenting morbidity in healthcare systems, encoding mortality statistics, and billing purposes. The ICD-11 encodes RDs more comprehensively and precisely. This paper refers to the ICD-10 and ICD-11 versions of the World Health Organization (WHO).	^83^
Orphanet Rare Disease Ontology (ORDO)	Structured open-access ontology for RDs that enables queries of rare disorders and captures relationships between diseases, genes, and other relevant features.	^84^
Systematized Nomenclature of Medicine Clinical Terms (SNOMED CT)	Most comprehensive, standardised and precise clinical health terminology providing codes, terms, synonyms, definitions, and relationships of concepts used in clinical documentation and reporting. For our RD-CDM v2.0, we utilised SNOMED CT Version 2024-09-01. However, it is important to note that SNOMED CT is not permitted in all countries due to licensing restrictions or varying adoption policies.	^85^
Logical Observation Identifiers Names and Codes (LOINC)	Widely used terminology for clinical and laboratory observations, health care screening instruments, and document type identifiers that provide a set of identifiers, names, and codes for a wide range of observations, focusing on laboratory tests. For our RD-CDM v2.0, we utilised LOINC Version 2.78.0.	^86^
Human Genome Organisation - Gene Nomenclature Committee (HGNC)	Approving unique symbols and names for human loci, including protein-coding genes, non-coding RNA genes, and pseudogenes.	^87^
Human Genome Variation Society (HGVS)	Offers guidelines for cataloguing variations in DNA, RNA, and protein sequences and recommends the adoption of HGNC gene symbols in their notation.	^88^
Online Mendelian Inheritance in Man (OMIM)	Comprehensive and authoritative catalogue focusing on the molecular relationship between genetic variation and phenotypic expressions containing information on all known Mendelian disorders and over 15,000 genes.	^89^
NCI Thesaurus OBO Edition (NCIT)	The National Cancer Institute Thesaurus is a reference terminology covering a broad domain of clinical findings, abnormalities, cancer, and cancer-related diseases.	^90^
NCBI organismal classification (NCBITaxon)	Organism names and classifications for all sequences in the nucleotide and protein databases of the International Nucleotide Sequence Database Collaboration (INSDC) are provided by the National Center for Biotechnology Information (NCBI) Taxonomy.	^91^
Units of Measurement Ontology (UO)	The Units Ontology (UO) provides a standardised description of units of measurement in science.	^92^
Evidence & Conclusion Ontology (ECO)	As a community resource, the Evidence and Conclusion Ontology (ECO) comprises ontology terms for the type of evidence supporting biomedical assertions and annotations.	^93^

**Table 3:** List of key organisations, data standards, and technologies used in the development of our RD-CDM with respective references (Ref.).

Name (Abbreviation)	Definition	Ref.
Health Level 7 (HL7)	International standards organisation that develops internationally applicable standards for the exchange, integration, sharing, and retrieval of electronic health information.	^21^
Fast Healthcare Interoperability Resources (FHIR)	Enables structured data exchange in the healthcare sector, enabling semantic and syntactic interoperability. We used and refer to version 4.0.1 of its base resources for this work.	^21^
Observational Medical Outcomes Partnership (OMOP) CDM	Defined by the Observational Health Data Sciences and Informatics (OHDSI) community, including internationally usable tools for data integration and analysis and several approaches to improve the display of rare diseases.	^17^
GA4GH Phenopacket Schema	An International Organization for Standardization (ISO) standard by the Global Alliance for Genomics and Health (GA4GH) defines a computable representation of clinical data that enables deep phenotyping, supports the analysis and exchange of complex phenotypic information, and facilitates the machine-readability and comparison of patient records. For this work, we used and refer to version 2.0.	^22^
ART-DECOR	Open-source tool suite that supports creating and maintaining data models to be implemented on various platforms linked to ontologies and terminologies to ensure interoperability and reusability.	^94^
Research Electronic Data Capture (REDCap)	Web- and survey-based application to capture data and create databases and projects for clinical research, freely available and adopted in many institutes worldwide.	^95^

A dataset is a collection of related and discrete data elements that can contain various data types, such as numerical or categorical data. Data modelling specifies how a dataset is organised within a database, defining the structure, collection methods, metadata, features and interconnections between data elements^23^. The European Rare Disease Registry Infrastructure Common Data Set (ERDRI-CDS) specifies data elements as a standardised basis for capturing RD data to enhance registry data reuse across Europe. Its eight sections, such as Patient Status or Care Pathway, include 16 data elements in total^24^. Since it serves as a basis for all RDs, it is limited in complexity and length, further elaborated in the Evaluation section^25^. The French Minimal Data Set for Rare Diseases (F-MDS-RD) is a standardised collection of data elements designed to support the collection and reporting of RD data nationwide. It was developed through a national consensus and is used in all French Rare Disease centres. The nationwide consensus allows a more integrated approach to data capture within healthcare information systems^20^.

## Results

The Results section is structured as follows: the RD-CDM section offers a detailed overview of the entire model and its components, followed by the Evaluation subsection, which is the most comprehensive part of the article. The Evaluation subsection is organised into three categories: (1) Medical Data Granularity, (2) Medical Reasoning and Clinical Relevance, and (3) Interoperability and Harmonisation. This structure is also used in the Discussion section for consistency and readability.

### RD-CDM

**Fig. 1:**
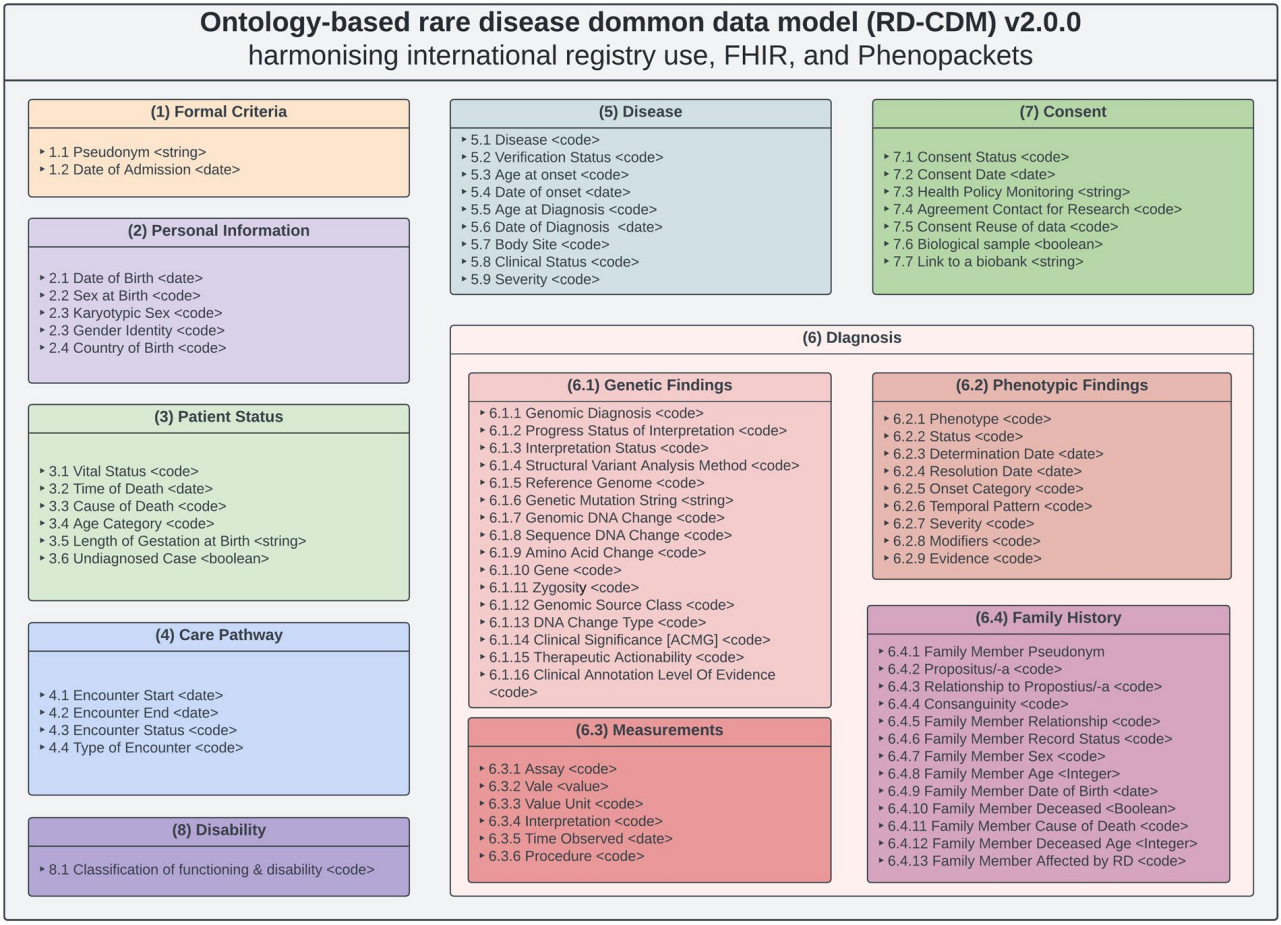
Depicts version 2.0.0 of our ontology-based rare disease common data model (RD-CDM) based on the European Rare Disease Registry Infrastructure - Common Data Set (ERDRI-CDS), HL7 FHIR base resources v4.0.1 and the GA4GH Phenopacket Schema v2.0. The sections are derived from the ERDRI-CDS, and the section Diagnosis is extended by four subsections. For each section, all data elements and their data types. This data model does not define any cardinalities of relationships between elements or sections.

Our data model counts 78 data elements, extending the ERDRI-CDS by 62 elements. All eight sections of the ERDRI-CDS remained, although section one was renamed to Formal Criteria, section five to Disease, and section seven to Consent. Section six, Diagnosis, was divided into four subsections: Genetic Findings, Phenotypic Findings, Measurements, and Family History. Figure 1 depicts the organisation of our RD-CDM without cardinalities or relationships defined. The diagram gives an overview of all sections and subsections, including the value types of all data elements.

**Fig. 2:**
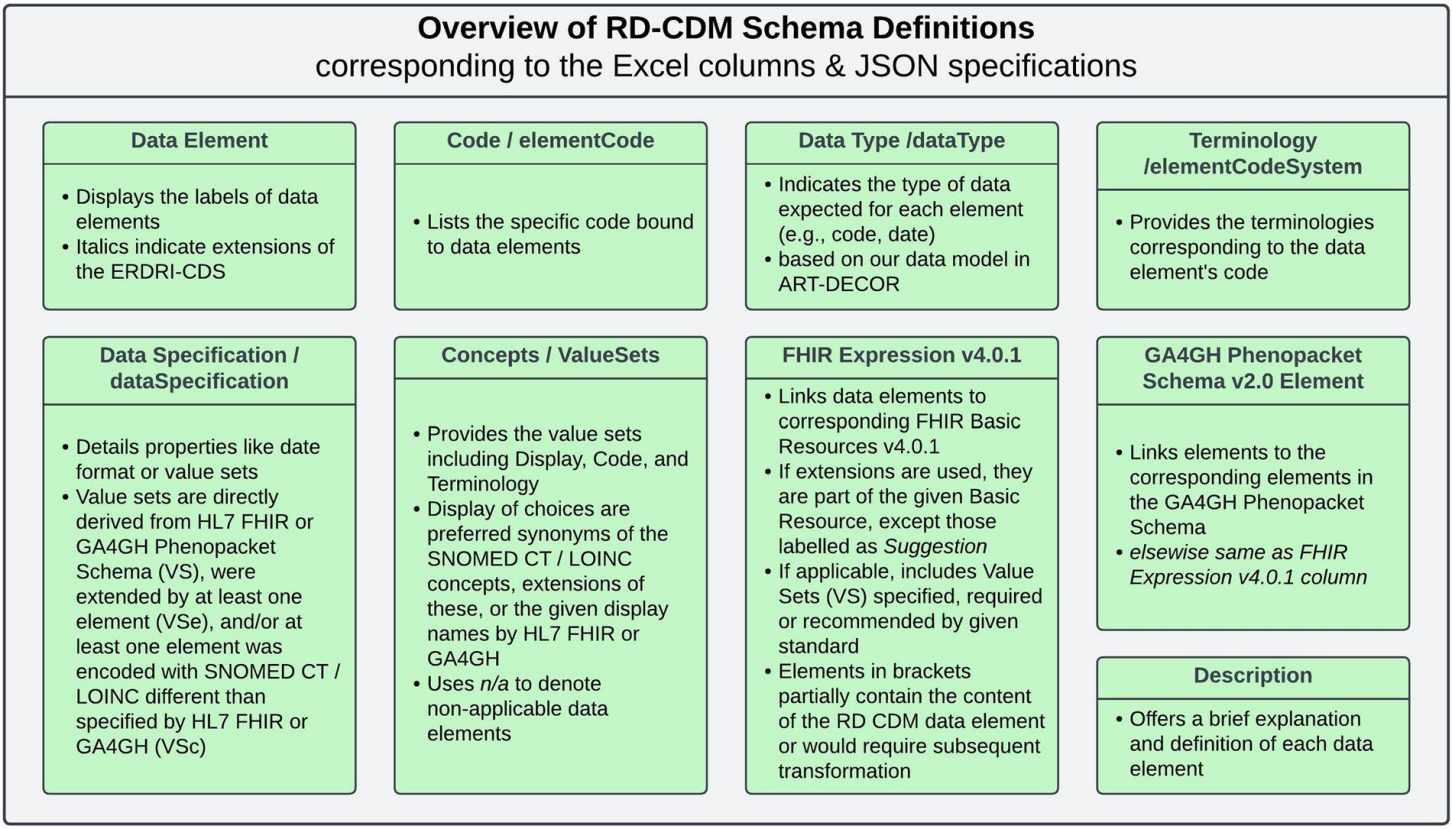
Provides an overview of the table columns and JSON specifications used to depict our rare disease common data model (RD-CDM). All abbreviations are established, and further definitions and explanations are given. We recommend referring to this figure when viewing the RD-CDM’s Excel Table or JSON files^12^.

The following paragraphs provide an overview of our data model as specified in the Excel sheet on Figshare^15^, our GitHub repository^16^ and ART-DECOR project^26^, detailing all data elements, terminology bindings, and value sets established through our work. The table columns are categorised to represent different aspects of the data model, depicted in Fig. 2. Each section in the table aligns with specific attributes of the data model, including variable codes, descriptions, and coding systems. These specifications defined the JSON schema for our current version, v2.0.0 of the RD-CDM^15^, ensuring a structured and machine-readable format. Both the JSON schema and the JSON files of our data model can be found in our GitHub repository and its documentation^16^.

The *Data Element* column depicts the label of each data element, while those in italics are extensions of the ERDRI-CDS. The *Code(s)* and *Terminology* columns list specific codes and their respective terminologies assigned to these elements. *Data Type* indicates the type of data expected (e.g., code, date). *Data Specification* elaborates on additional properties, such as the date format or value sets, which are either directly derived from HL7 FHIR or the GA4GH Phenopacket Schema (VS), where some value sets were extended (VSe) by at least one element, and/or at least one element was mapped to a different SNOMED CT, NCIT, HPO, or LOINC value (VSc) other than specified by HL7 FHIR or GA4GH. If applicable, *Concepts* provides a value set’s *Display*, *Code*, and *Terminology*. The displays of the value set choices are either the preferred synonyms of the SNOMED, HPO, or LOINC concept, extensions of these preferred synonyms, or the given names defined by the given Standard (GA4GH, HL7 FHIR). Elsewhere, *n/a* denotes that a data element is not applicable. The *FHIR Expression v4.0.1* and *GA4GH Phenopacket Schema Element v2.0* columns link to the corresponding FHIR Resources or elements within the GA4GH Phenopacket Schema. If applicable, the given standard’s recommended or required value sets (VS) are specified. All extensions used are part of the given HL7 FHIR base resource, except those labelled *Suggestion*, which we propose as potential extensions. Expressions in brackets represent elements that partially contain the content of our RD-CDM data element or would require subsequent transformation. Lastly, the *Description* column briefly explains and defines each data element.

This first section of our RD-CDM was renamed to *Formal Criteria* in contrast to the ERDRI-CDS section. The patient-related identification code was derived from the ERDRI-CDS and bound to the SNOMED CT (SCT) code *Patient-related Identification code* (SCT:422549004). Based on the FHIR resource *Patient*, this element corresponds to the *Individual.id* within the GA4GH Phenopacket Schema. Extending the ERDRI-CDS, we added the data element *1.2 Date of Admission* based on the FHIR element *Encounter.period.start* from the FHIR Encounter resource to depict the date of data capture or admission for specific use cases. Within the GA4GH Phenopacket Schema this data element can be expressed with the *Individual.time_at_last_encounter* element.

The second section (2) Personal Information contains the individual’s personal information. While the *2.1 Date of Birth* and *2.2 Sex at Birth* are included in the ERDRI-CDS, we added the individual’s *2.3 Karyotypic Sex*, *2.4 Gender*, and *2.5 Country of Birth* based on the FHIR resources *Observation* and *Patient*. The *2.5 Country of Birth*’s value set uses ISO3166 (International Standardization Organization) for 2- or 3-letter country codes as required by HL7 FHIR. Within the GA4GH Phenopacket Schema, data elements 2.1–2.4 are expressed within the *Individual* building block.

In section (3) Patient Status, the elements *3.1 General Clinical State* and *3.2 Time of Death* are derived from the ERDRI-CDS. These are linked to the FHIR resource *Patient* and GA4GH Phenopacket Schema block *Individual*. The ERDRI-CDS value set was extended with *Unknown* and encoded with SNOMED CT. We extended this section by the individual’s *3.3 Cause of Death* and *3.4 Age Category* corresponding to the *Patient* and *Observation* FHIR resources. *3.3* is associated with a diagnosis based on ICD-10 codes, while *3.4* was linked to an extensional SNOMED CT-based value set for the different periods of life. The element *3.5, Length of Gestation at Birth*, was added as a string defined with ‘XX + X’ for the weeks and days, respectively. Based on the ERDRI-CDS, the element *3.6 Undiagnosed RD Case* is a boolean indicating cases where an RD diagnosis has not been established, codable, for example, with the ORDO code 616874 (Rare disorder without a determined diagnosis after full investigation). Within the GA4GH Phenopacket Schema, all elements except 3.5 and 3.6 are found in the *Individual* block.

The only data element within the corresponding section of (4) Care Pathway in the ERDRI-CDS is called ‘First contact with a specialised centre’. We designed this section of our data model using the FHIR resource *Encounter*. For the data element *4.4 Type of Encounter*, we enhanced the HL7 FHIR value set linked to Encounter.class by adding a custom code, ‘rd_specialist_center’. This addition addresses the absence of the term ‘RD specialist’ in SNOMED, NCIT, HPO, or LOINC, where valid post-coordination is also not possible (further detailed in the Evaluation Section). Thus, combined with the data element *4.1 Encounter Start*, the date of the first encounter with a specialist centre can be expressed. We also expanded this section with elements *4.2 Encounter End* and *4.3 Encounter Status* with their respective HL7 FHIR value sets to allow the display of other patient encounters.

The section (5) Disease is based on the ERDRI-CDS section *5. Disease History* including its elements and value sets *5.4 Age at onset* and *5.6 Age at diagnosis* with respective elements for the date of onset and diagnosis (5.5 and 5.7). These were encoded with SNOMED CT and can be expressed using the FHIR resource *Condition* or partially the GA4GH Phenopacket element *Disease.onset*. We renamed this section to *(5) Disease* and extended it based on the HL7 FHIR resource *Condition*. This allows the encoding of multiple diseases with genetic diagnoses, subtypes (5.1) and verification statuses (5.2). Further, the disease’s body site, clinical status and severity (5.7–5.9) can be captured. Within the GA4GH Phenopacket Schema, these elements are mainly in the Disease block, except for the age at diagnosis (5.5) and severity (5.9).

Similar to the ERDRI-CDS, we divided the section (6) *Diagnosis* into four subsections but renamed them to *(6.1) Genetic Findings*, *(6.2) Phenotypic Findings*, *(6.3) Measurements*, and *(6.4) Family History*. For example, the element *Undiagnosed case* from the ERDRI-CDS is found under (3) Patient Status as element *(3.6)*. The *Family History* was added based on the HL7 FHIR *FamilyMemberHistory* resource and the GA4GH Phenopacket Schema *Pedigree* block.

Based on element *6.2, Genetic Diagnosis* in the ERDRI-CDS, we created element *6.1.1 Genomic Diagnosis*, that can be linked to a disease (5.1) if the same OMIM codes are used. Its status can be displayed using the required GA4GH Phenopacket Schema element *6.1.2 Progress Status of Interpretation*. Multiple variants can be linked to a specific disease, selecting the *6.1.3 Interpretation Status*. Each variant can be further described with the analysis method, its reference genome, a string, the HGVS expressions, the gene studied, and its zygosity (6.1.4–6.1.11). Further, we enriched each variant with the *6.1.12 Genomic Source Class*, *6.1.13 DNA Change Type*, *6.1.14 Clinical Significance (ACMG)*, and the *6.1.16 Clinical Annotation Level of Evidence* based on the HL7 FHIR resource *Observation* in Genomics v4.0.1. The element *6.1.15 Therapeutic Actionability* from the GA4GH Phenopacket Schema was added as required within the *VariantInterpretation* building block.

Section (6.2) Phenotypic Findings also extends beyond the ERDRI-CDS, though it maintains the recommended HPO coding for a *(6.2.1) Phenotypic Finding*. The element *(6.2.2) Status* was derived from the HL7 FHIR resource *Observation*, but its value set was simplified to *Confirmed present* and *Refuted* corresponding to the Phenopacket element PhenotypicFeature.excluded. Further, *(6.2.3) DeterminationDate and (6.2.4) Resolution Date* were added to our data model, alongside *(6.2.8) Modifiers* and *(6.2.9) Evidence*, which were all derived on the PhenotypicFeature Block in Phenopackets v2.0 and corresponding to the FHIR resource Observation. We also added the elements (6.2.5) Onset Category, (6.2.6) Temporal Pattern, and (6.2.7) Severity, all based on the Phenopacket’s PhenotypicFeature block and corresponding to the FHIR Observation resource. We defined HPO-based value sets for these three to enable categorical clustering within more detailed phenotyping.

Additionally, we added section (6.3), Measurements, based on the Phenopacket’s Measurement block. This section includes the elements *(6.3.1) Assay, (6.3.2) Value, (6.3.3) Value Unit, (6.3.4) Interpretation, (6.3.5) Time Observed*, and *(6.3.6) Procedure*, encoded with NCIT and SNOMED CT. These elements correspond to the FHIR resources Observation and Procedure.

The entire (6.4) Family History section is an extension of the ERDRI-CDS detailing the individual’s family history in our data model. RD-specific elements such as Propositus/-a, the relationship to the index case of consanguinity were added (6.4.2–6.4.4). All further elements were derived from the FHIR resources *FamilyMemberHistory*. For *6.4.5 Family Member Relationship* (and 6.4.3), we created a SNOMED CT-based value set for all natural family members based on the HL7 FHIR value set *FamilyMember*. The *deceasedBoolean* in the FHIR Resource *FamilyMemberHistory* was altered to an extended value set, including the option *Unknown*. In addition, the data element *6.4.13 Family Member Diagnosis* corresponds to the HL7 FHIR *FamilyMemberHistory.condition.code* element and GA4GH Phenopacket Schema element *Pedigree.Person.affectedStatus* while depending on the elements *5.1 Disease* and *6.1.1 Genomic Diagnosis*. Within the *Family.relatives* block of the GA4GH Phenopacket Schema, a list of separate Phenopackets can be created and linked by the individual ID (1.1) and the *6.4.1 Family Member Pseudonym* with most data elements in this section. *6.4.4 Consanguinity* and *6.4.7 Family Member Sex* can be expressed directly with GA4GH Phenopacket Schema elements.

Within our data model, section (7) Consent corresponds to the ERDRI-CDS section *7. Research* with its data elements *7.4 Agreement to be contacted for research purposes*, *7.5 Consent to the reuse of data, 7.6 Biological sample*, and *7.7 Link to a biobank*. While these specific elements have a limited depiction within SNOMED CT, element 7.3, for example, was bound to the LOINC concept *Research study consent* (77602-1). Further, we extended this section with the data elements *7.1 Consent Status*, *7.2 Consent Date*, and *7.3 Health Policy Monitoring* required within the FHIR resource *Consent*.

Section (8) Disability corresponds directly to the ERDRI-CDS section *Disability* with its data element *8.1 Classification of functioning/disability*. This element is encoded with our custom encoding *icf_score* as neither SNOMED pre- or post-coordination, NCIT, HPO, nor LOINC allows for the encoding of this element. Its value is encoded with the International Classification of Functioning, Disability and Health (ICF). Although the ICF is not represented in the GA4GH Phenopacket Schema, the HPO term *Impairment of activities of daily living* (HP:0031058) could be used.

### Evaluation

**Fig. 3:**
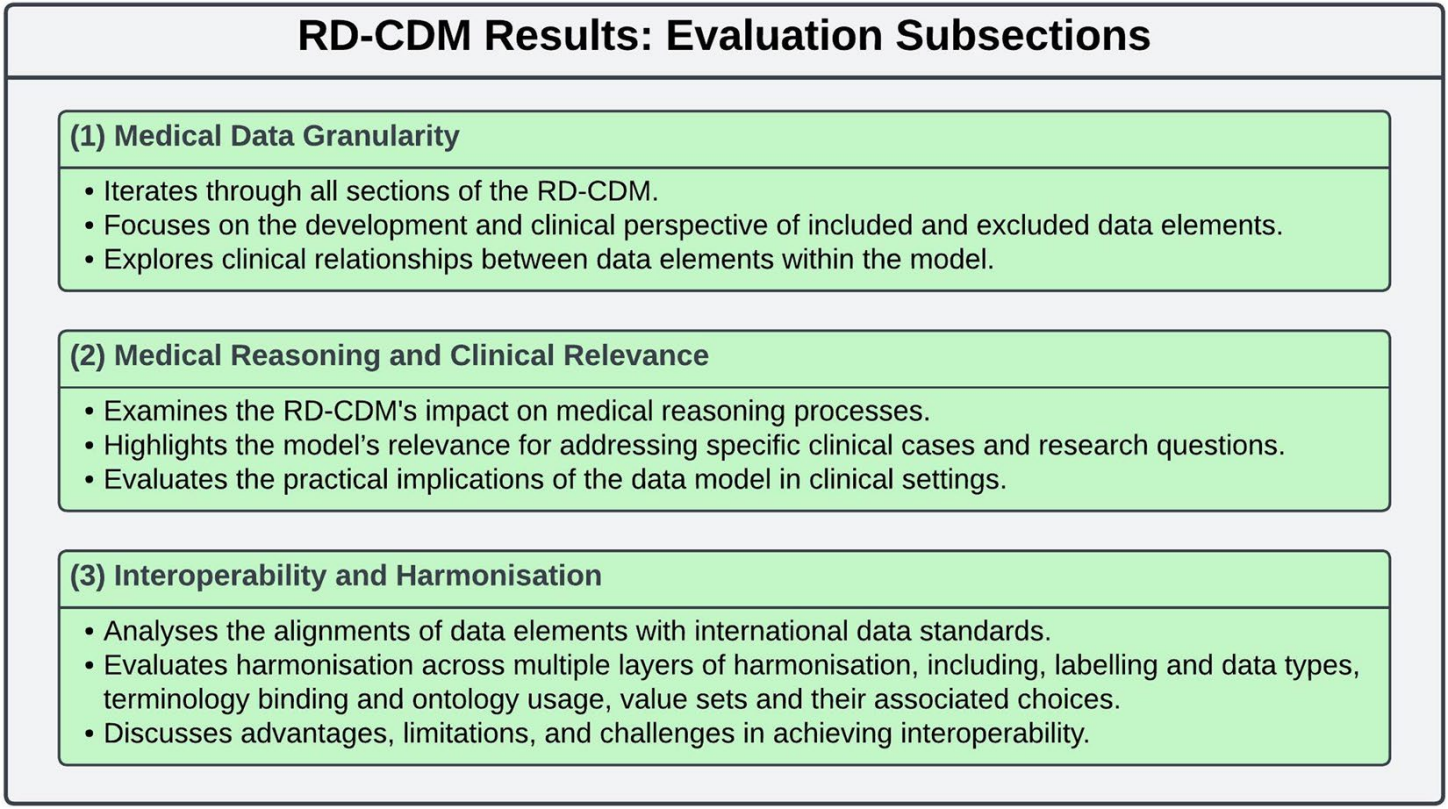
Illustrates the three evaluation categories used to assess the RD-CDM. (1) Medical Data Granularity evaluates each section of the model, focusing on the development and clinical perspective of data elements and their interrelationships. (2) Medical Reasoning and Clinical Relevance examines the RD-CDM’s impact on clinical decision-making and its applicability to specific clinical cases and research questions. (3) Interoperability and Harmonisation analyses the alignment of data elements with international standards, including labelling, data types, terminology binding, and value sets, while discussing advantages, limitations, and challenges in achieving interoperability. This structured evaluation provides insights into the clinical significance, limitations, and future challenges of the RD-CDM.

This subsection evaluates the development of our RD-CDM (Fig. 3), specifically the extension process of the ERDRI-CDS and challenges related to harmonisation and interoperability. The evaluation focuses on the development and extension process of the ERDRI-CDS while encompassing challenges related to harmonisation and interoperability. Implementing the proposed RD-CDM in the clinical settings of four university hospitals allowed for testing and further refinement.

Based on our methodology for developing our model, we identified the following three evaluation categories (Fig. 3). In (1) Medical Data Granularity, we iterate through all sections of our RD-CDM, each specifying the development and clinical perspective on the included data elements and their relationships. Based on these, we elaborate on our RD-CDM’s effects on (2) Medical Reasoning and Clinical Relevance. Lastly, we discuss the advantages, limitations and challenges of (3) Interoperability and Harmonisation while developing our RD-CDM. For this, we iterate through several layers of harmonisation, analysing the alignments of data elements with the data standards used, including their labelling, data types, terminology binding, and the value sets and their choices. This structured approach enabled us to comprehend the practical implications and clinical significance of each element within the data model, its limitations, and the challenges that need to be addressed in future.

### Medical data granularity

Registries are key in improving care and research for RD patients by connecting affected families, clinicians, researchers and clinical trials. A pseudonym or patient identifier (1.1) is often assigned locally and must always be captured to refer between several Phenopackets or further analyses. Although the identifier is context-dependent, it enables reidentification for studies or transferral of patient data after consent is given. We extended our RD-CDM with the *Date of Admission* (1.2), which is essential for registry submissions, categorising the individual’s *Age Category* (3.4), and establishing the temporal context of the individual’s *Care Pathway* (4) and *Disease* elements (5).

Besides the ERDRI-CDS elements *Date of Birth* (2.1) and *Sex at Birth* (2.2), we extended our data model with the individual’s *Karyotypic Sex* (2.3) and self-assigned *Gender* (2.4). This approach enables differentiation between an individual’s gender, sex assigned at birth, and karyotypic sex, reducing the likelihood of inaccurate entries and increasing the precision of data on sex and gender. Additionally, the differentiation between gender, sex at birth, and karyotypic sex can be relevant for conditions like gender dysphoria, which impacts gender identity as opposed to the sex assigned at birth^27–29^ or mixed gonadal dysgenesis affecting an individual’s sexual development^30^. Notably, the accuracy of the *Gender* element (2.3) may be subject to a bias and vary depending on an individual’s country of residence due to different political circumstances.

Although section *(2) Personal Information* was further extended with the individual’s *Country of Birth* (2.4), we excluded the individual’s ethnicity. While ethnicity-specific rare variants were associated with Alzheimer’s disease^31^ and other RDs, note an increased prevalence among specific ethnic groups, such as the extremely rare AADC Deficiency^32^, this element remains highly controversial. Additionally, ethnicity value sets often lack precision and do not display the increased amount of *mixed* people in migrating countries, risking promoting biases. Therefore, we aligned with the GA4GH’s Phenopacket Schema v2.0, which does not include an ethnicity element, and excluded it from the current of our RD-CDM. At present, the element (2.5) Country of Birth can serve as an indicator. An ethnicity data element may be considered for future versions. For now, we recommend that users conducting ethnicity-based clinical studies follow established guidelines^33^ for any extensions to our RD-CDM.

Linked with the *Date of Admission* (1.2), the patient’s *Vital Status* (3.1), and, if deceased, *Time of Death* (3.2) are vital for registry use or therapy studies. We extended the ERDRI-CDS value set with *Unknown*, as proposed by the GA4GH Phenopacket Schema. We have expanded our data model to include the ICD-10 encoded *Cause of Death* (3.3). Although this data can sometimes be imprecise^34^, it remains essential for understanding RDs as they often cause premature death^35,36^. The individual’s *Age Category* (3.4) can be linked to the *Date of Admission* (1.2) to facilitate more precise clustering in clinical or scientific studies, such as examining age-related diseases and pathologies^37^. Additionally, we enhanced our CDM with the *Length of Gestation at Birth* (3.5), which can aid in assessing the impacts of potential prematurity or post-maturity on health and discovering novel variants and diseases in individuals who are small for gestational age^38^. Lastly, the *Undiagnosed RD Case* (3.6) element was added to align with the ERDRI-CDS. Although this could be determined indirectly with the given data model, we kept this element to allow direct queries for registry purposes and linkage to ORDO-encoded diseases with no diagnosis^39^.

Patients with RDs require a more complex care framework encompassing clinical, psychological, nursing, and specialist centre perspectives. RD patients typically move through various care stations on their diagnostic journey, often spanning many years. This includes specialist visits, inpatient and ambulatory stays, and, frequently, home health care provided by parents or other relatives^4,7,40,41^. Thus, a complex display of a patient’s care pathway can be beneficial in understanding the patient’s clinical course. We have integrated the period times (4.1, 4.2), *Status* (4.3) and *Class* (4.4) of encounters based on the HL7 FHIR Encounter resource into our data model. This enables the documentation of multiple encounters throughout an individual’s lifespan. Further, this data can be linked with the *Disease* (5) and *Phenotypic Features (6.2)* sections to create a more comprehensive profile. As the ERDRI-CDS requires the time of the first encounter with an RD specialist centre, the *Encounter Class* (4.4) value set was enhanced with an *RD Specialist Center* choice that can be linked with the encounter’s start date (4.1).

In contrast to the ERDRI-CDS section *Disease History*, we have renamed this section to *(5) Disease* in our RD-CDM. Our extension based on the HL7 FHIR resource *Condition* provides a comprehensive overview of any number of diseases. The *Disease* element (5.1) allows multiple encoding with ORDO, ICD-10, ICD-11, MONDO, OMIM_g, and OMIM_p to encode disease subtypes and genomic diagnosis, which are increasingly important for RDs^10^. The *Date of Diagnosis* (5.6) and *Verification Status* (5.2) can differentiate between confirmed, excluded, or differential diseases over time. This can significantly enhance the diagnosis, understanding, and treatment of RDs^2,10,42^, such as idiopathic inflammatory myopathies^43^ or Familial Chylomicronemia Syndrome^44^.

Phenopackets are better suited for capturing the onset as phenotypic expressions rather than the timing of a medical diagnosis. The ERDRI-CDS elements *Age* and *Date at Onset or Diagnosis* (5.3–5.6) from the ERDRI-CDS can be linked with the section *(6.2) Phenotypic Findings* and *Date of Birth* (2.1) to determine the onset codes of the HPO concept *Onset* (HPO:0003674). This approach can be beneficial in case-level phenotyping for genomic diagnostics and discovery^45^. Additionally, we enhanced our RD-CDM with the elements *Body Site* (5.1.7), *Clinical Status* (5.1.8), and *Severity* (5.1.9) derived from the HL7 FHIR *Condition* resource. Although a disease’s severity may be imprecise due to individual clinical assessments and impractical for later analyses, the HL7 FHIR resource requires inclusion.

Section *6.1 Genetic Findings* enables the comprehensive display of multiple variants that can be linked with a *Genomic Diagnosis* (6.1.1), and if the same OMIM codes are used, also with a specific *Disease* (5.1). While the ERDRI-CDS recommends using HGVS and HGNC codes, we differentiate between an unvalidated HGVS string (6.1.6), validated genomic, sequence and protein level HGVS expressions (6.1.7–6.1.9) and the HGNC code for the gene studied (6.1.10). Therefore, if medical professionals are uncertain during data capture, 6.1.6 allows for an initial recording of all genetic information with subsequent validation. When our CDM is employed within healthcare information systems, we recommend the integration of automated HGVS validators^46^ to increase accuracy for RD diagnostics and research^10^.

All further elements of (*6.1) Genetic Findings* section were based on the GA4GH Phenopacket Schema, the HL7 FHIR resource Observation in Genomics v4.0.1 and the HL7 FHIR Genomics Reporting profiles v3.0.0 Variant and DiagnosticImplication. The mandatory GA4GH’s *Progress Status of Interpretation* (6.1.2) and *Therapeutic Actionability* (6.1.15) enhance the variant’s diagnostic and therapeutic implications. Although therapies are excluded in the current version of our RD-CDM, a variant’s actionability could support therapy-specific extensions to the model. Utilising the variant’s *Interpretation Status* (6.1.3), multiple *causative* variants can be linked to a single genomic diagnosis to account for dual Mendelian diagnoses^47^. Besides the *Reference Genome* (6.2.5) and *Zygosity* (6.2.11), each variant is enhanced with its *Genomic Source Class* (6.2.12), *Change Type* (6.2.13), *Clinical Significance* according to ACMG (6.2.14), and the *Clinical Annotation Level of Evidence* (6.2.16), enabling a more profound understanding and clinical evaluation of the variants. However, the rating of the genetic variant could change over time if new information becomes available (e.g. data derived from functional characterisation), highlighting the need for precise and thorough documentation of phenotypic features^48^. Nevertheless, our data model’s extensive genetic findings section can enable complex genotype-phenotype correlation analyses and machine-learning-based approaches. This advancement aims to enhance our understanding of RDs, ultimately improving clinical predictions, disease management, and symptom control.

Besides diagnoses, a comprehensive overview of an individual’s symptoms is essential for RD research and care to enable deep phenotyping^9^. In contrast to the ERDRI-CDS^24^, we have enhanced our model by extending HPO-encoded phenotyping (6.2.1) independent of a disease verification status (5.2) and have further enriched this data with its *Status* (6.2.2) and *Determination Date* (6.2.3) and *Resolution Date (6.2.4)*, respectively. While a phenotypic feature can be observed and recorded at different times, we recommend using the observed time by the individual or clinician for *6.2.2*. The exclusion of specific symptoms over time can support the exclusion of diseases to diagnose a range of RDs^42–44^. Further, a longitudinal display of symptoms and measurements is essential in understanding and treating RDs, such as rare kidney diseases^49^ or monogenic obesity^50^.

Additionally, we included phenotypic *Modifiers* (6.2.8) to further describe a specific phenotypic feature by using clinical modifiers (HP:0012823) or linking causative infectious agents using the NCBITaxon Ontology. The *Evidence* (6.2.9) can be relevant in identifying differences between, e.g., patient-reported outcome measurements and phenotypes determined by medical staff^51,52^. Furthermore, we created HPO-based value sets for the *Onset Category (*6.2.5), the *Temporal Pattern* (6.2.6), and the *Severity* (6.2.7), contributing to case-level phenotyping within cohorts^45^ and potentially enhancing complex analysis algorithms^53^. Further, we have added a Measurements section (6.3) as laboratory data and other measurements can play a key role in diagnosing and understanding many rare diseases^10,49,50,54^.

We have expanded our model with the section *Family History* to enable a comprehensive overview of family patterns since over 70% of RDs have a genetic cause^1^ and can be beneficial for registry use. With the identification of a *Propositus/-a* (6.4.2), their *Relationship to the Index Case* (6.4.3) or *Consanguinity* (6.3.4) family pedigrees can be constructed to understand inheritance patterns. Additionally, consanguinity is vital since it can elevate the risk of recessive genetic disorders^55^. Further, our CDM allows a comprehensive display of biological relatives (6.4.5), such as entry *Status, Sex, Age, and Date of Birth (6.4.6*–*6.4.9)*. If a family member is *Deceased* (6.4.10), the *Cause of Death* (6.4.11) and *Deceased Age* (6.4.12) can be captured. Through the element *Family Member Disease* (6.4.13), we also provide the capability to indicate whether the documented family member is affected by the same RD as the individual (5.1). To accommodate situations where details are uncertain, we have included the *Unknown* choice for many value sets, acknowledging that many individuals have incomplete yet valuable information, such as knowing some relative was affected and died at a certain age.

### Medical reasoning and clinical relevance

The implementation in a clinical setting, capturing real patient data for registry use and analysis, allowed us to develop, evaluate and contextualise our RD-CDM. From a clinical perspective, defining how and with what weighting individual clinical and genetic findings are converted and summarised into data elements remains unclear. This is due to the wide variation in each specific use case’s implementation, aims, goals, and clinical context. Based on our experience, the limitations and rationale behind this conversion should be carefully examined to enhance its effectiveness for each use case. Thus, we identified several issues and pitfalls related to medical reasoning that must be addressed to improve the RD-CDM’s clinical relevance for medical professionals, patients, and researchers.

To enhance suitability in a clinical setting, we aligned our data model with the logical flow of medical history to potentially increase the amount of reusable data captured. For example, we included *Unknown* values in many fields since patients are often unaware of specific details. A phenotypic finding’s status can be confirmed or refuted, providing clarity, and can be enhanced with clinical modifiers to indicate severities or infectious agents, offering greater flexibility. Additionally, we retained the HGVS string element (6.1.6) to allow for the inclusion of genomic interpretations and findings without prior validation.

Similar to previous approaches harmonising rare disease databases^56^, we experienced diversity in data quality, particularly concerning the FAIR principles and machine-readability. The lack of standardised terms for clinical characteristics or symptoms, varying reference values, and the absence of clear definitions for complex clinical pictures pose a significant challenge for secondary data use, making the translation of patient data into the CDM a crucial step. From our experience, this translation depends on multiple factors, including the specific use case, its implementation into the healthcare information system, and the semantic structure of the baseline data model. While the full implementation of our RD-CDM into REDCap remains a focus for future work, we have addressed several issues within our data model to minimise semantic errors and improve data quality. This includes reducing the number of free-text data elements wherever possible, developing ontology-based value sets for consistency between cohorts, and limiting the extension of the ERDRI-CDS to elements derived from either FHIR base resources or the Phenopacket Schema. Additionally, implementing the model within a public GitHub repository alongside documentation^16^ has proven essential for enabling flexible updates and extensions and improving usability, semi-automation, and bug-fixing for external users.

Our RD-CDM has proven feasible for trial use in clinical settings, maintaining its core validity for multiple RDs and semantic consistency. Enabling both registry export and precise analyses was regarded as highly valuable by our participating clinicians and rare disease researchers. However, its limited scope is both an advantage and a limitation. Considering the limited time and resources available for RD research, the limited length of our RD-CDM allows more straightforward implementation and, eventually, automated data capture. In contrast, our current version of RD-CDM does not cover many medical data elements relevant to RDs, such as therapy-specific data. While these can be added in future versions, this limitation restricts the current scope of its medical reasoning capabilities.

Regarding medical reasoning within RDs, the duck test in data science can be applied. It suggests that if something looks, swims and sounds like a duck but has batteries beneath, you likely have the wrong abstraction. In the context of RDs, this can showcase the diagnostic process - finding the right abstraction and asking the right question to discover and diagnose an RD^57^. As our RD-CDM is limited in scope, the risk of biases within RDs is increased by restricting the potential perspectives and abstractions to find *the batteries*. Clinically, this bias is often evident in human genetics, where variant-based clinical predictions are frequently imprecise due to the scarcity of high-quality data on the clinical course and disease progression of RDs^58^. In contrast, this bias is also counteracted by our RD-CDM, as its alignment with international standards can significantly increase the amount of precise clinical course data available. This raises an inherent paradigm, both limiting and extending its semantic capabilities, that must be carefully considered when utilising an RD-CDM and potential disease-specific extensions.

### Interoperability and harmonisation

**Fig. 4:**
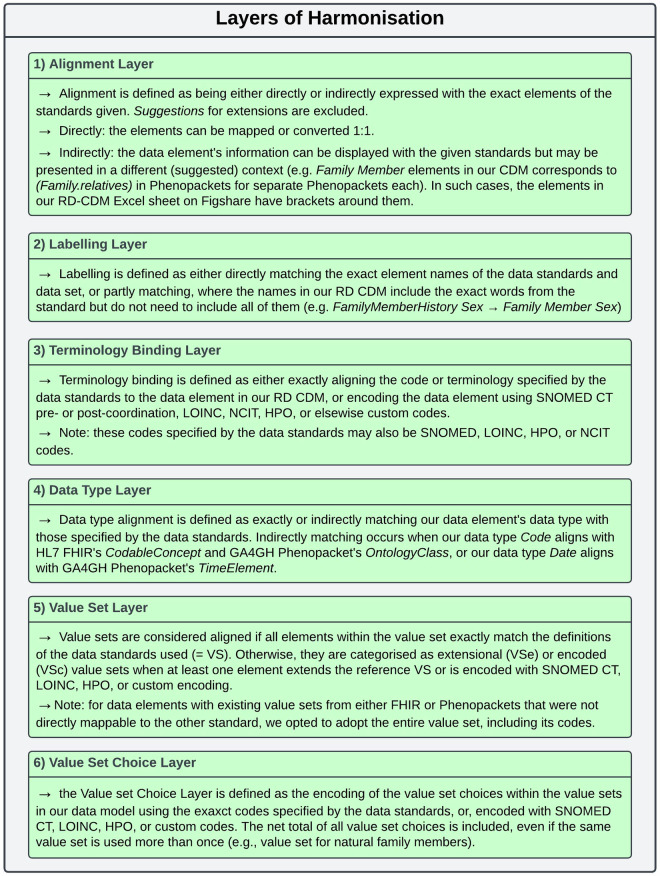
Depicts the definitions of the layers of harmonisation that were identified upon development and evaluation of our RD-CDM. Further, the selection criteria for each layer are specified, which were applied to Tables 4–9. In some cases, examples are given to depict indirect criteria for the layers defined, or notes are provided to clarify potential misunderstandings. We recommend viewing this figure when reading through the tables and text of this section.

This section analyses to what extent interoperability requirements were met while harmonising data elements from the ERDRI-CDS, HL7 FHIR base resources and the GA4GH Phenopacket Schema to a single RD-CDM. For this, we identified six layers of harmonisation on the level of each data element: (1) the Alignment Layer, (2) the Labelling Layer, (3) the Terminology Binding Layer, (4) the Data Type Layer, (5) the Value Set Layer, and (6) the Value Set Choice Layer. All layers and their selection criteria are depicted in Fig. 4. For each layer, we provide a detailed account of every data element in each section, quantifying the number of elements in our RD-CDM that conform to the specifications of data models and standards used, as depicted in the Tables 4–9. We evaluate the advantages, challenges, and limitations of our approach to utilising SNOMED CT and LOINC, NCIT, and HPO as common denominators for our RD-CDM. Further, we critically reflect on the limitations, implications, and advantages of our methodology in adhering to interoperability requirements for RD research and care.

**Table 4:** Depicts the alignment of data elements of our RD-CDM v2.0.0 to HL7 FHIR Resources v4.0.1, the GA4GH Phenopacket Schema v2.0, both HL7 FHIR Resources and the GA4GH Phenopacket Schema, or neither.

1) Alignment Layer					
	Data elements aligned to FHIR	Data elements aligned to the Phenopackets Schema	Data elements aligned to FHIR and the Phenopackets Schema	Data elements aligned to neither FHIR nor the Phenopackets Schema	∑(RD-CDM)
(1) Formal Criteria	—	—	1.1, 1.2	—	2
(2) Personal Information	2.5	—	2.1–2.4	—	5
(3) Patient Status	3.5	—	3.1–3.4, 3.6	—	6
(4) Care Pathway	4.1–4.4	—	—	—	4
(5) Disease	5.5, 5.9	—	5.1.–5.4, 5.6–5.8	—	9
(6.1) Genetic Findings	6.1.4, 6.1.12–6.1.14, 6.1.16	6.1.2, 6.1.3, 6.1.15	6.1.1, 6.1.5–6.1.11	—	16
(6.2) Phenotypic Findings	—	—	6.2.1–6.2.9	—	9
(6.3) Measurements	—	—	6.3.1–6.3.6	—	6
(6.4) Family History	6.4.6	6.4.2–6.4.4	6.4.1, 6.4.5, 6.4.7–6.4.13	—	13
(7) Consent	7.1–7.5	—	—	7.6, 7.7	7
(8) Disability	—	—	—	8.1	1
Sum	19	6	50	3	78
% of all elements	24%	8%	64%	4%	100%

Alignment is defined as being either directly or indirectly expressed with the exact elements of the standards given. It shows that 24% of all 78 data elements align with HL7 FHIR resources, 8% align with the GA4GH Phenopacket Schema, 64% align with both HL7 FHIR resources and the GA4GH Phenopacket Schema, and 4% align with neither.

**Table 5:** Depicts the derivation of data element labelling for our RD-CDM v2.0.0, which aligns either with the ERDRI-CDS, HL7 FHIR v4.0.1 base resources or the GA4GH Phenopacket Schema v2.0, or neither, where a custom name was assigned.

2) Labelling Layer				
	Data element label derived from the ERDRI-CDS	Data element label derived from HL7 FHIR or the Phenopacket Schema	Data element label derived from neither (custom label)	∑(RD-CDM
(1) Formal Criteria	1.1	—	1.2	2
(2) Personal Information	2.1, 2.2	2.3, 2.4	2.5	5
(3) Patient Status	—	—	3.1–3.6	6
(4) Care Pathway	—	4.1–4.4	—	4
(5) Disease	5.3, 5.5	5.2, 5.7–5.9	5.1, 5.4, 5.6	9
(6.1) Genetic Findings	—	6.1.1–6.1.16	—	16
(6.2) Phenotypic Findings	6.2.1	6.2.2–6.2.9	—	9
(6.3) Measurements	—	6.3.1–6.3.6	—	6
(6.4) Family History	—	6.4.1, 6.4.5–6.4.12	6.4.2–6.4.4, 6.4.13	13
(7) Consent	7.4–7.7	7.1–7.3	—	7
(8) Disability	8.1	—	—	1
Sum	11	52	15	78
% of all elements	14%	67%	19%	100%

Labelling is defined as the data element’s names either directly or partly matching with those of the data standards or set or given. The table highlights that 67% of data element labelling is derived from HL7 FHIR Resources or the GA4GH Phenopacket Schema, 14% from ERDRI-CDS, and 19% are assigned custom names.

**Table 6:** Illustrates the terminology binding, defined as aligning the code or terminology specified by the data standards HL7 FHIR or the GA4GH Phenopacket Schema to the data element in our RD-CDM v2.0.0.

3) Terminology Binding Layer					
	Data element’s code according to HL7 FHIR (may also be LOINC/SNOMED/HPO/NCIT)	Data element’s code according to GA4GH Phenopacket Schema	Data element encoded with with SNOMED CT, LOINC, NCIT or HPO	… custom codes	∑(RD-CDM)
(1) Formal Criteria	—	—	1.1, 1.2	—	2
(2) Personal Information	—	—	2.1–2.5	—	5
(3) Patient Status	—	—	3.1–3.6	—	6
(4) Care Pathway	4.1, 4.2, 4.4	—	4.3	—	4
(5) Disease	—	—	5.1–5.9	—	9
(6.1) Genetic Findings	6.1.4–6.1.10, 6.1.12–6.1.14, 6.1.16	6.1.2, 6.1.3, 6.1.15	6.1.1, 6.1.11	—	16
(6.2) Phenotypic Findings	—	6.2.9	6.2.1–6.2.8	—	9
(6.3) Measurements	—	—	6.3.1–6.3.6	—	6
(6.4) Family History	6.4.6	—	6.4.2–6.4.5, 6.4.7–6.4.13	6.4.1	13
(7) Consent	7.2	—	7.1, 7.3, 7.6	7.4, 7.5, 7.7	7
(8) Disability	—	—	—	8.1	1
Sum	16	4	53	5	78
% of all elements	21%	5%	68%	6%	100%

Alternatively, it involves encoding using SNOMED CT pre- or post-coordination, LOINC, NCIT, HPO, or, if not possible, custom codes. The table shows that 21% of all 78 data elements align with HL7 FHIR resources, 5% with the GA4GH Phenopacket Schema, 68% were encoded with either SNOMED CT, LOINC, NCIT, or HPO, and 6% with custom codes. Notably, 10 out of the 16 data elements encoded according to HL7 FHIR, and none of the 4 data elements encoded according to the Phenopacket Schema, are also encoded with terminology codes (either LOINC, SNOMED, NCIT, or HPO). This represents 80.77% (n = 63) of the data elements encoded with one of the specified terminologies.

**Table 7:** Depicts the data type binding of data elements in the RD-CDM v2.0.0 with the given standards used.

4) Data Type Layer					
	Data element value types according to HL7 FHIR resources	Data element value types according to the GA4GH Phenopacket Schema	Data element value types according to HL7 FHIR Resources & the GA4GH Phenopacket Schema	Data element value types according to neither HL7 FHIR Resources nor the GA4GH Phenopackets Schema	∑(RD-CDM)
(1) Formal Criteria	1.1	—	1.2	—	2
(2) Personal Information	2.5	2.3	2.1, 2.2, 2.4	—	5
(3) Patient Status	3.4	—	3.1–3.3	3.5, 3.6	6
(4) Care Pathway	4.1, 4.2, 4.3, 4.4	—	—	—	4
(5) Disease	—	—	5.1–5.9	—	9
(6.1) Genetic Findings	6.1.4, 6.1.12, 6.1.13, 6.1.16	6.1.2, 6.1.3, 6.1.15	6.1.1, 6.1.5–6.1.11, 6.1.14	—	16
(6.2) Phenotypic Findings	—	—	6.2.1–6.2.9	—	9
(6.3) Measurements	—	—	6.3.1–6.3.6	—	6
(6.4) Family History	6.4.1, 6.4.5, 6.4.6, 6.4.11	—	6.4.7, 6.4.9, 6.4.13	6.4.2–6.4.4, 6.4.8, 6.4.10, 6.4.12	13
(7) Consent	7.1–7.5	—	—	7.6, 7.7	7
(8) Disability	—	—	—	8.1	1
Sum	20	4	43	11	78
% of all elements	26%	5%	55%	14%	100%

Within FHIR resources, a *CodableConcept*, or in the GA4GH Phenopacket Schema, an *OntologyClass*, is equivalent to the data type *Code* in our model. Further, a *TimeElement* in the GA4GH Phenopacket Schema is equivalent to a *Date* value type in our model. The table categorises data elements based on their value type alignment with HL7 FHIR resources, GA4GH Phenopacket Schema, both HL7 FHIR resources and GA4GH Phenopacket Schema, or neither. It shows that 26% of all 78 data element value types align with HL7 FHIR resources, 5% with GA4GH Phenopacket Schema, 55% with both HL7 FHIR resources and GA4GH Phenopacket Schema, and 14% with neither.

**Table 8:** Describes the value set layer for data elements in the RD-CDM v2.0.0.

5) Value Set Layer					
	Value Sets according to HL7 FHIR Resources	Value Sets according to the GA4GH Phenopacket Schema	Value Sets extended (VSe) and/or encoded (VSc) with SNOMED/LOINC/HPO	Value Sets extended (VSe) and/or encoded (VSc) with mixed codesystems	∑(RD-CDM)
(1) Formal Criteria	—	—	—	—	0
(2) Personal Information	—	—	2.2–2.4	—	3
(3) Patient Status	—	—	3.1, 3.4, 3.6	—	3
(4) Care Pathway	4.3	—	—	4.4	2
(5) Disease	5.3, 5.8–5.10	—	5.4, 5.6	—	6
(6.1) Genetic Findings	6.1.4, 6.1.6, 6.1.12, 6.1.13, 6.1.16	6.1.2, 6.1.3, 6.1.15	6.1.11, 6.1.14	—	10
(6.2) Phenotypic Findings	—	—	6.2.2, 6.2.5–6.2.7	—	4
(6.3) Measurements	—	—	—	—	0
(6.4) Family History	6.4.6	—	6.4.2–6.4.5, 6.4.7, 6.4.10	—	7
(7) Consent	7.1	—	7.4, 7.5, 7.6	—	4
(8) Disability	—	—	—	—	0
Sum	12	3	23	1	39
% of all elements	31%	8%	59%	2%	100%

Value sets are only considered as aligned with HL7 FHIR or GA4GH Phenopacket Schema if the entire value set, including its data element’s encoding, were exactly derived from these standards. Otherwise, they are categorised as extensional (VSe) or encoded (VSc) value sets when at least one element is added and/or encoded with SNOMED CT, LOINC, or HPO. The table categorises data elements based on their value set’s alignment with HL7 FHIR Resources, GA4GH Phenopacket Schema, and extended value sets encoded with SNOMED CT, LOINC, or HPO. It shows that from all 39 value sets in our RD-CDM, 31% align with HL7 FHIR and 8% with the GA4GH Phenopacket Schema. Extended value sets comprise more than half of all values, with 59% encoded with SNOMED CT, LOINC, or HPO and one value set (2%) with mixed codes. Notably, we opted to adopt the entire value set, including its codes, for data elements with existing value sets from either FHIR or Phenopackets that were not directly mappable to the other standard.

**Table 9:** Describes the value set choice layer for the data element’s value sets in the RD-CDM v2.0.0, where the net total of all value set choices is included, even if the same value set is used more than once (e.g., the value set for natural family members).

6) Value Set Choice Layer							
	Value Set Choices in encoded with HL7 FHIR codes	Value Set Choices in encoded with GA4GH Phenopacket Schema codes	Value Set Choices in encoded with SNOMED CT	Value Set Choices in encoded with LOINC	Value Set Choices in encoded with HPO	Value Set Choices in encoded with Custom Codes (RD-CDM v2.0.0)	∑(RD-CDM)
(1) Formal Criteria	—	—	—	—	—	—	0
(2) Personal Information	—	—	21	—	—	—	21
(3) Patient Status	—	—	15	—	—	—	15
(4) Care Pathway	15	—	1	—	—	1	17
(5) Disease	16	—	11	—	—	—	27
(6.1) Genetic Findings	37	13	—	13	—	—	63
(6.2) Phenotypic Findings	—	—	2	—	23	—	25
(6.3) Measurements	—	—	—	—	—	—	0
(6.4) Family History	4	—	40	—	—	—	44
(7) Consent	6	—	9	—	—	—	15
(8) Disability	—	—	—	—	—	—	0
Sum	78	13	99	13	23	1	227
% of all elements	34%	5%	45%	5%	10%	1%	100%

The value sets are categorised based on their alignment with HL7 FHIR v4.0.1, GA4GH Phenopacket Schema v2.0, SNOMED CT, LOINC, HPO, and custom encoding. The table shows that 34% of the value set choices align with HL7 FHIR, 5% with GA4GH Phenopacket Schema, 45% use SNOMED, 5% use LOINC, 10% use HPO, and 1% use custom codes.

In the following, we iterate through the layers of harmonisation to evaluate how many elements of our RD-CDM comply with the data standards used (HL7 FHIR & GA4GH Phenopacket Schema). Out of all 78 data elements in our RD-CDM v2.0.0, 75 (96%) are aligned with either HL7 FHIR resources or the GA4GH Phenopacket Schema (Table 4). Regarding their labelling, 14% (n = 11) of elements are labelled according to the ERDRI-CDS, 67% (n = 52) according to either HL7 FHIR or GA4GH Phenopackets, and 19% (n = 15) are custom labels (Table 5). This corresponds to our methodology to use the ERDRI-CDS as a basis for our RD-CDM while developing all its data elements and extensions in alignment with HL7 FHIR and the GA4GH Phenopackets Schema.

Regarding the Terminology Binding Layer, 6% (n = 5) of data elements are defined with custom codes. In comparison, 68% (n = 53) are bound to either LOINC, SNOMED CT, HPO, or NCIT codes, and 26% (n = 20) are bound to codes either specified by HL7 FHIR or the GA4GH Phenopacket Schema (Table 6). In comparison, 86% (n = 67) of the data elements’ data types are directly or indirectly those specified by either HL7 FHIR or GA4GH Phenopacket Schema, and 14% (n = 11) are not (Table 7). Out of these 11 data elements, three are ERDRI-CDS elements (7.6, 7.7, 8.1), five are novel RD-specific elements (3.5, 3.6, 6.4.2–6.4.4), two have an integer data type in our model, but Age value type required in HL7 FHIR (6.4.8, 6.4.12), and one data element should be a boolean, which we extended to a value set to include the option *unknown* (6.4.10). This reflects the difficulties in harmonising RD-specific elements, among others, from the ERDRI-CDS with interoperability data standards.

Of all 39 value sets, 38% (n = 15) were directly derived from HL7 FHIR resources or the GA4GH Phenopacket Schema. Out of the extended or encoded value sets (VSe/VSc), 58% (n = 23) were encoded with SNOMED CT, LOINC, or HPO, and one (2%) was mixed with HL7, SNOMED CT and custom encoding (4.4). Of these value sets, there is a net total of 227 value set choices, out of which 40% (n = 91) are encoded according to codes specified by HL7 FHIR or GA4GH Phenopacket Schema. In total, 59% of value set choices (n = 135) are encoded with either SNOMED, LOINC, or HPO codes, and one with a custom code (1%), which is part of the Encounter Class value set (4.4). This custom code is the RD specialist centre, which is not codable yet in any of the standards or terminologies used ‒ but is required by the ERDRI-CDS.

Notably, we adopted the entire existing value set, including its codes, for data elements with existing value sets from either FHIR or Phenopackets that were not directly mappable to the other standard. If comparing the value set layer with the data type layer, meaning an encoded choice for a CodeableConcept or an OntologyClass, we find that 87% of value sets (n = 34) are aligned with the specifications required by either FHIR and/or Phenopackets. Value sets of elements (6.4.2), (6.4.3), (6.4.4), (7.6), and (6.4.10) are not directly aligned with the data type from these standards. These must be converted from a code to a Boolean (6.4.10) or could not be directly mapped to either HL7 FHIR v4.0.1 or the Phenopacket Schema v2.0 (elements 6.4.2–6.4.4, 7.6). The latter four are more complex RD-specific concepts required by the ERDRI-CDS, for which extensions will be necessary for the HL7 FHIR v4.0.1 standard.

Data reusability is crucial for improving efficacy and efficiency in RD research and care while addressing inherent data scarcity and distribution challenges. To achieve this, our RD-CDM integrates three key components: the ERDRI-CDS, the HL7 FHIR standard, and the GA4GH Phenopacket Schema. However, to achieve semantic interoperability, all systems and models should use the same code.

This paradigm is informed by data standards like HL7 FHIR, which developed codes for use but are not always consistently aligned with international ontologies or terminologies. To address discrepancies between codes defined or suggested by HL7 FHIR and those required for RDs, we extended these using SNOMED CT, LOINC, NCIT, and HPO where feasible. This approach was selected to define a common semantic denominator for elements in our RD-CDM to bridge between HL7 FHIR, the GA4GH Phenopacket Schema, the ERDRI-CDS, and other RD models^17,20^. However, this strategy has several advantages, limitations, and future implications.

Ontologies are based on dependencies and relationships to each other, enhancing precision and analysis potential. Previously, ontology-based approaches were used to design healthcare system architectures^59^ or to link different communication standards^60^. Correct versioning remains instrumental, ensuring consistency between data standards and integrated models. In other settings, real-time data streams may translate more efficiently to ontologies than HL7 FHIR resources^61^ or improve knowledge-based retrieval in specific HL7 FHIR implementations^62^.

In contrast, semantic interoperability is not always ensured natively with HL7 FHIR or the GA4GH Phenopackets Schema, making a translation necessary. Further, SNOMED is not used in all countries worldwide, while HL7 FHIR and GA4GH Phenopackets can be implemented everywhere. A data model that specifies new codes instead of utilising those defined by the standards used aggravates the challenge of achieving semantic interoperability throughout the entire healthcare domain. While our approach helped encode RD-specific data elements, we had to custom code three data elements (6.4.1, 6.4.3, 8.1) and one value set choice (4.4) because neither SNOMED, LOINC, NCIT, nor HPO codes matched perfectly. Although SNOMED CT post-coordination was possible for one code (6.2.3), it is highly susceptible to errors and did not suffice for the custom-coded concepts. Despite our intention to officially register these SNOMED CT post-coordinated and custom codes, we modelled a data model valid for all RDs. Therefore, if disease-specific extensions are developed, SNOMED CT, LOINC, NCIT, and HPO may not suffice, requiring further updates within these codesystems.

As a non-balloted extension of the ERDRI-CDS, our RD-CDM could help streamline balloted CDMs based on international interoperability standards required for RD research and care^12^. When implemented in various healthcare information systems, our ontology-based approach could also enhance the development of standardised transformation pipelines to generate HL7 FHIR resources or GA4GH Phenopackets, improving syntactic interoperability. However, when implemented in other countries for various use cases, further collaboration and feedback are required to ensure operational flexibility and reliable semantics of our RD-CDM.

Our RD-CDM, at this stage, has no implementation guide and therefore no defined cardinalities. An Implementation Guide is crucial as it provides structured instructions and constraints to ensure consistent, interoperable, and accurate implementations across systems. Therefore, upon implementation of the current RD-CDM, cardinalities must be defined for specific use cases. In this evaluation’s Medical Data Granularity section, we recommended which sections should be repeatable, which must be included, and which may be optional. In technical terms, a 0…1 relationship means there can be zero or one instance of the related entity, a 0…n relationship allows for zero or more instances, and a 1…1 relationship requires exactly one instance. Depending on the use case, we recommend conferring sections *(1) Formal Criteria* and *(7) Consent* with a 1…1 cardinality, and sections *(2) Personal Information, (3) Patient Status*, and *(8) Disability* with a 0…1 cardinality. In contrast, sections *(4) Care Pathway, (5) Disease, (6.1) Genetic Findings, (6.2) Phenotypic Findings, (6.3) Measurements*, and *(6.4) Family History* should be conferred with 0…n relationships to allow multiple entries upon single data entry of an individual.

While the flexibility of our RD-CDM v2.0.0 is enhanced by not defining cardinalities, this can limit syntactic, organisational and technical interoperability due to varying implementations across systems and sites. Although such adherence is essential for the success of an RD-CDM^12^, its conceptual framework and GitHub repository^16^ may be better suited for updates, extensions, and further development. Potentially, our model could serve as a baseline model for country-specific versions, independent of healthcare information systems or regional constraints. However, this highlights the need to test our RD-CDM further in diverse clinical settings, systems, and sites to continue refining our model.

## Discussion

Clinical care and research for rare diseases (RDs) face persistent challenges, including the inherent diversity of over 10,000^3^ diseases with distinct requirements and the lack of data interoperability between cohorts, registries, and institutions. While registries like the European Reference Networks, based on the ERDRI-CDS, are essential for connecting patients, clinicians, and researchers, they often do not fully integrate international interoperability standards. International data standards like HL7 FHIR^21^ enable the accurate transfer of routine clinical data, while the GA4GH Phenopacket Schema supports case-level phenotyping^45^ and advanced analyses through its ontology-based approach, capturing both detailed clinical and genetic information^22^. The RD-CDM introduced in this paper can enhance the quality and precision of RD data capture by harmonising the ERDRI-CDS, FHIR and the Phenopackets Schema using embedded ontologies and terminologies. With 78 data elements, the ERDRI-CDS has been extended by 62 additional elements while maintaining its primary structure. By harmonising these standards, our model specifically aims to address and balance these distinct use cases by integrating them within a single model. This integration can enable seamless data capture for clinical, research, and registry purposes, positioning the Phenopacket Schema for broader use in the European Reference Networks and bridging the gap between routine care and specialised RD data. To maintain a consistent structure with the paper’s subsections, the discussion will also iterate through the Methods and the three evaluation subsections: Medical Data Granularity, Medical Reasoning and Clinical Relevance, and Interoperability and Harmonisation.

The non-hierarchical methodology in this work integrates the quality-assured inclusion, encoding, mapping, clinical evaluation, and trial implementation of our model’s data elements. This process is supported by open-source repositories and documentation^16^, supporting the sustainable development and deployment of our RD-CDM across various settings, systems, and regions. Consistency and compatibility with existing data frameworks may be improved, eventually enhancing the development of FHIR and Phenopacket interfaces, modules, and open-source tools. These insights and methodologies can also be adapted for other projects, Domain-Specific Common Data Elements^13^, or extensions around our model, such as the therapy section in the F-MDS-RD^20^. By providing a standardised approach to data integration and interoperability, collaboration in RD research can be enhanced.

Evaluating the Medical Data Granularity as part of the inclusion and exclusion criteria was a central step in the development of our model. Although the model’s length and complexity were limited to facilitate implementation, achieving the level of detail required for specialist-level research across all RDs proved challenging. The collaboration with clinical experts from diverse rare disease domains was essential. Each element was iteratively reviewed to define its clinical context for specific cases or research questions, ensuring meaningful relationships between elements. While the resulting version of our RD-CDM does not claim to be the perfect solution, it provides a foundational baseline for broader adoption. We acknowledge the need for continuous clinical evaluation as RD research and implementation progress, which may lead to the inclusion or exclusion of elements in future versions.

Discussing the Medical Reasoning and Clinical Relevance helped contextualise our findings for RDs. Although this paper is not a clinical study, the implementation in clinical settings allowed us to evaluate the development of our RD-CDM from a clinical perspective in real-life situations. Further, this approach helped us maintain clinical semantics throughout specific use cases, proving our model’s clinical significance. However, the conversion of specific clinical or genetic elements into the model remains unclear, indicating the variety of requirements to be considered upon use-case-specific implementation and the need for standardised guidelines for an implemented model. Also, the inherently limited scope of a CDM raises a paradigm in medical reasoning^57^, both enhancing and limiting semantic capabilities that may require close consideration upon use-case-specific implementation.

The evaluation of Interoperability and Harmonisation identified several implementation-specific errors and challenges, requiring further refinement, testing, and consensus-driven development. While over 96% of all data elements in the current version are directly aligned with FHIR or Phenopackets, around 85% of value types match the exact specifications defined by these standards. Regarding the terminology binding of data elements, over two-thirds of elements were encoded with either LOINC, SNOMED, HPO, or NCIT codes, around 25% with codes specified by either FHIR or Phenopackets, three with custom codes and one with a SNOMED CT post-coordinated code. We defined the rule to adopt the entire value set and its codes from either FHIR or Phenopackets if the respective data element could not be mapped to the other data standard. Thus, only around 60% of value sets and choices were encoded with the selected ontologies. However, comparing the value set layer with the data types, more than 87% of value sets are directly aligned with the specifications required by FHIR or Phenopackets. The remaining elements and value sets were mostly elements by the ERDRI-CDS or required subsequent conversion to be expressed in either one of these standards. Although these values may be reasonable at this stage, future developments in harmonisation, ontologies, and real-world implementations are expected to improve the harmonisation outcomes in future RD-CDM versions significantly.

These results highlight the challenge of harmonising the ERDRI-CDS, FHIR, and Phenopackets into a unified data model. They also emphasise the need for extensions within these standards and the selected ontologies to adequately display RD-specific elements necessary for registry use. Although the ontology-based approach limits semantic interoperability with FHIR, it can serve as a common denominator across multiple data standards and models. Additionally, this approach can enhance compatibility and reusability with existing data frameworks and increase the semantic capabilities of complex ontology-based analyses by leveraging the relationships defined within the ontologies.

The modular GitHub repository and documentation^16^ address the identified challenges by enabling wide usage, compatibility, and consistency across RD-CDM versions while preserving flexibility for updates, extensions, and bug fixes. This adaptability supports evolving standards and use cases, including disease-, system-, or country-specific requirements, as well as the development of modular and flexible data handling mechanisms. Furthermore, this flexibility can also be applied to other healthcare domains, allowing for the customisation of data models and their implementation in diverse healthcare information systems. This enables the establishment of system-specific guidelines while maintaining alignment with international standards. Although the data model definition is based on a custom schema, future updates will explore adopting more advanced frameworks, such as LinkML^63^, to enhance interoperability, compatibility, and functionality across frameworks.

Our findings align with international efforts and requirements to improve data management for RDs^12^. Unlike previous efforts^17–20,64,65^, our model harmonises HL7 FHIR, the GA4GH Phenopacket Schema, and the ERDRI-CDS utilising ontologies and terminologies. While the Phenopacket Schema natively adopts any ontology to enable profound analysis^22^, other analysis standards, such as OMOP, were not natively designed to adopt all RD-specific ontologies and concepts necessary for advanced RD analysis^66^. Although excluded from our RD-CDM, several approaches exist for aligning FHIR and OMOP^67,68^, which could facilitate data transfer and leverage OMOP-based analysis tools^17^.

The results indicate that our RD-CDM could be valuable for further international RD research, supporting comprehensive and consensus data collection according to FAIR principles. Our model could facilitate various applications, from local clinical use to federated analyses and international registry integration. As a non-balloted extension of the ERDRI-CDS, the RD-CDM v2.0.0 could be aligned with other balloted international versions. Future iterations could focus on further validation and implementation, enhancing our recommended cardinalities in other international healthcare information systems and for disease-specific use cases. Although the undefined cardinalities in our RD-CDM may limit the syntactic, organisational, and technical interoperability, it increases flexibility for implementation in any healthcare information system. Based on our experience, especially (semi-)automated processes were identified as essential to improve the model’s impact upon implementation.

For future implementations, development around the FHIR International Patient Summary^69^ and Genomics Reporting^70,71^ could create FHIR resources aligned with the European Health Data Space^72^. Based on our project in GitHub^16^ and ART-DECOR^26^, using integrated scenarios and FHIR Questionnaires^73^ may also be feasible. Interoperability-based frameworks in open-source systems such as REDCap, including automated data capturing, validation, and export mechanisms, should be investigated further while accommodating the opposing dependencies of marginal costs versus benefits^12^. This could enable clinicians worldwide to maximise the precision of captured data based on international standards, regardless of financial resources, coding skills, or geographical location. Full-scale and federated analyses could help refine our model and ensure precise semantics on a wider scale. Future research could also explore using large language models (LLMs) to increase the amount of interoperable data when a specific data model is integrated. Like ours, feeding an LLM with a standardised CDM could allow it to encode information from unstructured data sources using our specified codes and value sets.

The current version, 2.0.0, of our RD-CDM^15^ can be found in our GitHub repository and its documentation^16^, on Figshare^15^ and our public ART-DECOR project^26^ for further implementation and development. Any RD researcher, clinician, and technician is invited to collaborate in establishing feedback mechanisms and improving our model’s capabilities. Our approach could sustainably improve RD care and research by supporting international, open-source development and providing a methodology and foundation for creating country-specific RD-CDMs aligned with interoperability requirements. This, in turn, can increase the reusability of rare disease data across cohorts and countries for multiple use cases through close alignment with FHIR, Phenopackets, and international registries such as the European Reference Networks.

## Methods

**Fig. 5:**
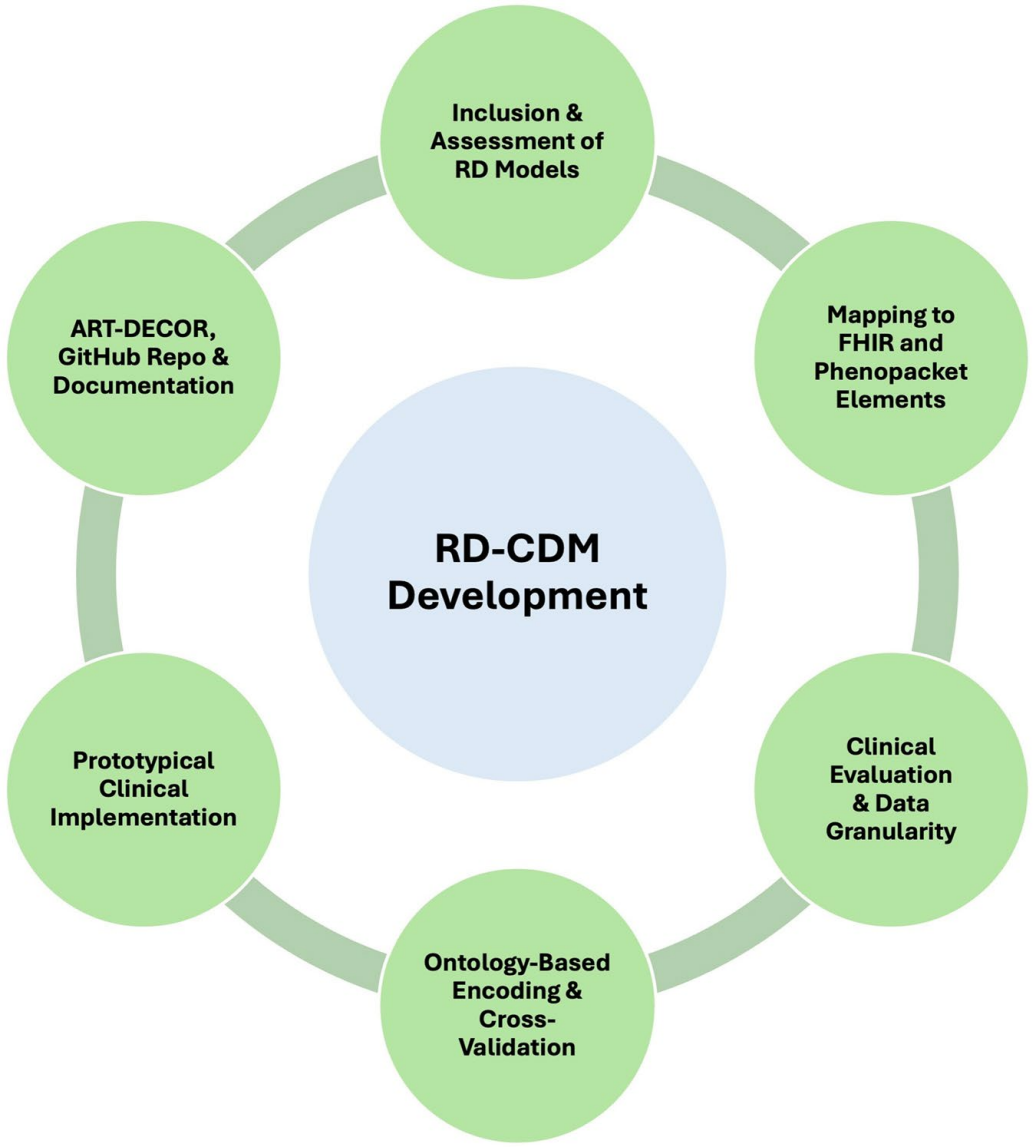
Steps in the development of the ontology-based rare disease common data model (RD-CDM) harmonising international registry use, FHIR and Phenopackets. The process was non-hierarchical, with overlapping steps across multiple sites. It involved assessing previous RD models, mapping elements to FHIR v4.0.1 and Phenopacket v2.0, clinical evaluation, ontology-based encoding and cross-validation, and prototypical implementations in REDCap using real patient data. The project was further supported by development in ART-DECOR and an open-source GitHub repository alongside documentation to ensure sustainability and reusability.

This section outlines the methodology and rationale behind the development and evaluation of our RD-CDM, a process involving several iterative and overlapping steps, as depicted in Fig. 1. As many steps were performed concurrently and overlapped across multiple sites, this methodology should be considered a non-hierarchical approach. First, we included and assessed previous RD data models, followed by mapping elements to FHIR base resources v4.0.1^74^ and Phenopacket Schema v2.0 elements^22^. A clinical evaluation was performed to evaluate the relevance of these elements while balancing the data model’s scope and spectrum of data granularity. We then performed ontology-based encoding to establish a common denominator between the models and data standards. Prototypical versions of our RD-CDM were implemented in REDCap, capturing real patient data from various RDs and use cases. Additionally, the project was developed in our public ART-DECOR project^26^ and open-source GitHub repository alongside its documentation^16^ to ensure sustainability, reusability and flexibility for future improvements and usage (see Fig. 5).

The ERDRI-CDS^24^ was selected as a basis for our RD-CDM^15^, including all its elements to comply with the minimal requirements for the European Reference Networks^14^. All of its eight main sections, such as Personal Information, Care Pathway, and Diagnosis, were adopted in our model. We renamed sections one to Formal Criteria, five to Disease, and seven to Consent. The section (6) Diagnosis was divided into four subsections: (6.1) Diagnosis, (6.2) Phenotypic Findings, (6.3) Measurement, and (6.4) Family History. We specifically drew inspiration from the F-MDS-RD^20^ and considered other models^17–19,64,65^, our previous requirement analysis^12^ and a conceptual analysis^12,75^.

All overlapping elements, particularly those from the ERDRI-CDS and F-MDS-RD, were semantically mapped to the elements of FHIR base resources v4.0.1^74^, the HL7 FHIR Genomics Reporting profiles v3.0.0 Variant and DiagnosticImplication^70^, and the Phenopacket Schema v2.0^22^. Any elements that could not be mapped were excluded, as we established a rule that all data elements extending ERDRI-CDS must align with at least one of these standards. To ensure full compatibility, all other necessary elements of the relevant FHIR resources or Phenopacket building blocks were included for further evaluation.

We evaluated these elements in collaboration with our clinical coauthors for medical relevance while balancing our model’s scope and data granularity. The clinical reasoning and discussions on each element are further detailed in the Evaluation Subsection on Medical Data Granularity. Our RD-CDM was designed to be globally applicable across all RDs, balancing complexity for detailed analyses with simplicity for ease of deployment. In modelling our data model, we prioritised enabling complex genotype-phenotype correlations, measurements, disease data, and the comprehensive display of patient histories while ensuring the connection to existing registries. However, to keep the RD-CDM^15^ concise, we excluded several data elements and medical areas in the current version that, while clinically relevant for RDs, would have extended the model significantly. For example, therapy or medication data elements were excluded from the current version but may be incorporated in future updates.

To establish a common semantic denominator, we adopted an ontology-based approach to harmonise the ERDRI-CDS, FHIR, Phenopackets, and other data sets in our RD CDM^15^. This ensures consistent semantic meaning and the utilisation of their inherent relationships for downstream analyses while supporting reliable FHIR-based data exchange between institutions or the European Reference Networks^14^. Additionally, the Phenopacket Schema^22^ allows for precise analysis of genetic data alongside clinical information, including phenotypes and measurements, thereby enhancing the reusability of registry data.

Wherever possible, we encoded all data elements and value set choices using LOINC version 2.7.8, NCIT version 24.04e, HPO version 2024-08-13, and SNOMED CT version 2024-10-01 in the RD-CDM version v2.0.0^15^. The code systems selected for specific elements or value sets are either recommended by the applicable data standards or are focused explicitly on the HPO for phenotypic findings, the NCIT for measurements, and SNOMED CT for all other elements. To ensure the quality of the selected codes, ontology encoding and mapping to FHIR and Phenopackets was carried out by multiple coauthors with clinical, bioinformatics, and medical informatics backgrounds, cross-validating each element at least three times. Additionally, we adopted the entire value set, including its codes, for data elements with existing value sets from either FHIR base resources or the Phenopacket Schema that were not directly mappable to the other standard.

We implemented multiple prototypical versions of the RD-CDM^15^ in REDCap, adhering closely to the FAIR principles. All REDCap variables and value sets were encoded according to our data model. Although cardinalities require definition, our RD-CDM v2.0.0^15^ does not specify cardinalities or implementation guides, allowing flexibility across different countries and use cases. The RD-CDM data capture sheets were uploaded in the local REDCap instance of four German university hospitals. Retrospective real-world patient data from various rare diseases was captured manually and semi-automatically from patient records or existing tabular databases, facilitated by import scripts and data entry personnel. While the specifics of the REDCap implementation and use case-specific data capture are subjects of future work, this implementation supported the development of our RD-CDM. It enabled its use for Phenopacket-based analyses and in European, German, and local registries. This process helped refine our data model, identify missing elements, resolve logical errors, outline implementation-specific recommendations and balance the inclusion of precise semantics for analysis while maintaining a streamlined model.

The RD-CDM was developed in a public ART-DECOR project^26^, an open-source tool suite that supports creating and maintaining data models, and an open-source GitHub repository with an MIT license^16^. Our modular, object-oriented API generates and validates CSV and JSON files of the RD-CDM to support its implementation into any hospital information system. The JSON files are generated and validated using the JSON-2020 Schema definition^76^ and our custom definitions, corresponding to the Excel version of our RD-CDM^15^ and Fig. 2. All functions, classes, and scripts were designed to be compatible with future RD-CDM versions, allowing for flexible extensions and updates. Additionally, we developed a Sphinx-based documentation^77^ (https://rd-cdm.readthedocs.io/en/latest/), which includes detailed information on the RD-CDM, downloadable resources, usage guidelines, contribution details, licensing, and a changelog. This setup facilitates our model’s adoption, implementation and co-development in diverse settings, enabling future versions or extensions.

Based on our methodology and the culmination of over two years of cross-institutional collaboration and development, we identified three key categories to evaluate our RD-CDM in its current state, further specified in the Evaluation Subsection (Fig. 3). First, our model’s (1) Medical Data Granularity was assessed from a clinical perspective, focusing on the rationale behind the inclusion and exclusion of every data element while iterating through all its sections. Second, we evaluated (2) Medical Reasoning and Clinical Relevance, examining how well the model addresses these aspects. Finally, (3) Interoperability and Harmonisation were analysed, and our ontology-based approach’s advantages, limitations, challenges, and implications were discussed. This involved iterating through six layers of harmonisation (Fig. 4): alignment, labelling, terminology binding, value types, value sets, and value set choices for all data elements concerning the standards and models used. We also evaluated the impact of this approach for specific implementation scenarios and provided recommendations for defining cardinalities in healthcare information systems.

## Data Availability

The rare disease common data model (RD-CDM) is available in our GitHub repository (https://github.com/BIH-CEI/rd-cdm), our documentation (https://github.com/BIH-CEI/rd-cdm), the standard-enabling platform ART-DECOR in German and English (https://art-decor.org/ad/#/erker-/datasets/dataset/2.16.840.1.113883.3.1937.777.87.1.1/2024-05-29T22:55:10) and in the Figshare repository 10.6084/m9.figshare.26509150.

## Code availability

Version 2.0.0 of our ontology-based rare disease common data model is available in our GitHub repository (https://github.com/BIH-CEI/rd-cdm), our documentation (https://github.com/BIH-CEI/rd-cdm), our public ART-DECOR project (https://art-decor.org/ad/#/erker-/project/overview) and the Figshare repository (10.6084/m9.figshare.26509150).
